# Chronic Wasting Disease in Cervids: Implications for Prion Transmission to Humans and Other Animal Species

**DOI:** 10.1128/mBio.01091-19

**Published:** 2019-07-23

**Authors:** Michael T. Osterholm, Cory J. Anderson, Mark D. Zabel, Joni M. Scheftel, Kristine A. Moore, Brian S. Appleby

**Affiliations:** a Center for Infectious Disease Research and Policy, University of Minnesota, Minneapolis, Minnesota, USA; b Prion Research Center, Colorado State University, Fort Collins, Colorado, USA; c Minnesota Department of Health, Saint Paul, Minnesota, USA; d National Prion Disease Pathology Surveillance Center, Case Western Reserve University, Cleveland, Ohio, USA; VA Palo Alto Health Care System

**Keywords:** chronic wasting disease, infectious disease, prion disease, prions, public health

## Abstract

Chronic wasting disease (CWD) is a prion-related transmissible spongiform encephalopathy of cervids, including deer, elk, reindeer, sika deer, and moose. CWD has been confirmed in at least 26 U.S. states, three Canadian provinces, South Korea, Finland, Norway, and Sweden, with a notable increase in the past 5 years. The continued geographic spread of this disease increases the frequency of exposure to CWD prions among cervids, humans, and other animal species. Since CWD is now an established wildlife disease in North America, proactive steps, where possible, should be taken to limit transmission of CWD among animals and reduce the potential for human exposure.

## Abstract (precis)

Chronic wasting disease (CWD) is a prion-related transmissible spongiform encephalopathy of cervids, including deer, elk, reindeer, sika deer, and moose. CWD has been confirmed in at least 26 U.S. states, three Canadian provinces, South Korea, Finland, Norway, and Sweden, with a notable increase in the past 5 years. The continued geographic spread of this disease increases the frequency of exposure to CWD prions among cervids, humans, and other animal species.

## PRION-RELATED TSEs: LESSONS FROM THE PAST

The past century has provided compelling evidence that effective public health interventions are needed to prevent the transmission of prion-related transmissible spongiform encephalopathies (TSEs) between animals and humans and between humans. Creutzfeldt-Jakob disease (CJD), described initially in 1920, was the first recognized human TSE. CJD occurs in three forms: sporadic (85% of cases), hereditary (10% to 15% of cases), and acquired (usually through medical procedures [<1% of cases]) (1). While CJD has not been shown to transmit via casual contact, at least 492 iatrogenic infections have been documented since its recognition, prompting creation of medical guidelines for infection prevention (2). More than 95% of these infections were due to administration of prion-contaminated human growth hormone and gonadotropins and cadaveric dura mater grafts, with the rest a result of corneal transplants, reuse of contaminated neurosurgical instruments, and blood transfusions (in the case of variant CJD [vCJD]) (2). Identification of these risk factors has allowed the medical community to establish best practices related to TSE diseases, which have markedly reduced the risk of additional iatrogenic cases.

Kuru, first recognized in the 1950s and similar to CJD, was a major public health problem of the Fore tribe in Papua New Guinea decades before the advent of the prion hypothesis. The Fore people practiced ritualistic cannibalism, consuming the tissues of recently deceased family members as a sign of respect and mourning. Presumed to have originated following the consumption of a tribal member infected with sporadic CJD, kuru infected more than 2,700 individuals from the 1950s to the early 2000s (3, 4). About 60% of those infected were women, as they more often consumed the brain tissue, which contained the highest concentration of the infectious agent (5). The Australian government banned cannibalism among the Fore people in the 1950s, and the infection rate dropped. However, a number of cases occurred decades after the ban because of kuru’s long incubation period, which can exceed 50 years (4).

Bovine spongiform encephalopathy (BSE), or “mad cow disease,” is a recent example of the human health risk when TSEs occur in animals that enter the human food supply. The origin of BSE, which was first identified in cattle in 1986, is subject to speculation. BSE is thought to have either sporadically occurred in cattle, or it was introduced after cattle were fed meat-and-bone meal containing scrapie-contaminated sheep by-products (6). Regardless of origin, BSE cases increased substantially as a result of feeding calves BSE-contaminated, bovine-sourced meat-and-bone meal. The vast majority of BSE cases were identified in the United Kingdom, where nearly 1,000 cases were confirmed each week during the epidemic’s peak. Case numbers sharply declined after feeding of any ruminant tissue or ruminant by-product to cattle was prohibited. Other public health measures included permanently removing specified risk materials and tissues from the human food chain, BSE testing of cattle over 30 months of age before human consumption, strengthening BSE surveillance programs, and banning the movement of high-risk materials (6). However, an estimated 750,000 BSE-infected cattle entered the human food supply before these interventions (7).

The consequence of delayed interventions to curb the threat of BSE became evident in 1996, when the first case of vCJD was identified in a human (8). Epidemiologic and laboratory evidence has demonstrated that human cases of vCJD were related to consumption of BSE-contaminated beef. More than 200 cases of vCJD have been identified, with an average incubation of 10 years. Unlike sporadic CJD, which typically affects older individuals, the median age of onset for vCJD cases is 28 years (8). Also, concern remains that individuals with certain genotypes might still develop the disease or experience subclinical infections (9). This possibility warrants maintaining robust human surveillance to identify and mitigate any lingering vCJD threat. The BSE saga provides a compelling example of what can happen following human exposure to prions of animal origin.

Scrapie, first described hundreds of years ago, is a TSE that affects sheep and goats. Currently, there is no evidence of scrapie causing infections in humans. Despite that fact, substantial time and resources have been spent trying to eliminate scrapie from U.S. sheep and goats. The National Scrapie Eradication Program (NSEP), which was started in 2002, uses animal identification and slaughter surveillance to fight the disease. Since the program’s conception, scrapie prevalence in the country has decreased by 99% (10). The NSEP, which identified scrapie as an economic and food security threat, is a prime example of a successful preventive approach to help control an animal-associated TSE.

## CHRONIC WASTING DISEASE: BACKGROUND

**FIG 1:**
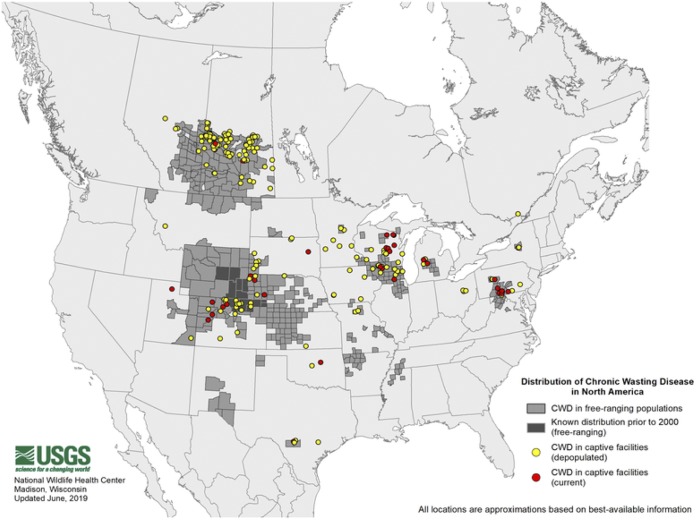
The distribution of detected chronic wasting disease cases in North American captive and free-ranging cervids as of June 2019, courtesy of the United States Geological Survey (https://www.usgs.gov/media/images/distribution-chronic-wasting-disease-north-america-0).

Chronic wasting disease (CWD) is a TSE that affects cervids, including deer, elk, reindeer, sika deer, and moose. It was first identified in 1967 in a captive mule deer living in a Colorado research facility. The increasing CWD disease risk in cervids is evident from surveillance data; in 2000, CWD was documented in five U.S. states and one Canadian province, in 2010, it was identified in 17 states and two provinces, and in 2018, it was found in 26 states and three provinces (Fig. 1). CWD has also been documented in South Korea, Finland, Norway, and Sweden. The disease is most likely transmitted horizontally through infectious bodily fluids such as saliva, urine, and feces (11). Once excreted into the environment, CWD prions can persist for years (12, 13) and withstand extremely high levels of disinfectants such as heat, radiation, and formaldehyde (14). CWD prions also appear to be capable of binding to certain plants, with the ability to be transported while still potentially remaining infectious (15).

CWD is increasing in cervids as more animals come into contact with infectious prions, usually via direct contact with an infected cervid and its bodily fluids, although viable CWD prions in the environment can also infect animals (14). As more cervids become infected, the frequency of these exposures and subsequent environmental contamination grows. Transmission among cervids is further complicated by continued allowance of risk factors known to propagate CWD. Factors such as baiting or feeding that promote the congregation of animals, improper carcass disposal, and anthropogenic movement of carcasses and live cervids play a key role in the continued spread of CWD (16). For example, the movement of infected live cervids between game farms has been identified as the cause of CWD introduction to South Korea. Movement of infected live cervids has also been implicated in the spread of CWD among North American deer and elk farms, with 10 states (Iowa, Michigan, Minnesota, Missouri, Montana, New York, Ohio, Oklahoma, Pennsylvania, and South Dakota) and three provinces (Alberta, Quebec, and Saskatchewan) detecting their first CWD cases in captive cervids. Additionally, circumstantial evidence suggests this movement may have led to infections among wild cervids in Saskatchewan, Nebraska, South Dakota, and Wisconsin (16). As a result of ongoing transmission among cervids, the frequency of human exposure to CWD prions is likely growing. The Alliance for Public Wildlife estimates that 7,000 to 15,000 CWD-infected animals are consumed annually, a number that may increase by 20% each year (17).

## LABORATORY TESTING AND DIAGNOSIS OF PRION-RELATED TSEs

At this time, two validated diagnostic assays with notable limitations are available to determine if a cervid is infected with CWD prions. Enzyme-linked immunosorbent assay (ELISA) and immunohistochemistry (IHC) are postmortem tests that detect the presence of abnormal prion proteins in the obex area of the brain stem or the retropharyngeal lymph nodes. ELISA testing has not been validated in elk or moose, so samples collected from these animals are confirmed via IHC. Valid ELISA and IHC testing for CWD can be conducted only in federally approved laboratories that are part of the National Animal Health Laboratory Network, so results often take days to weeks. This delay is significant, since hunters might choose to process or consume the meat from cervids in the interim. This could lead to unrecognized and unnecessary exposure to CWD prions and contamination of meat-processing equipment, as prions have been shown to bind to metal without losing infectivity (14). Additionally, ELISA and IHC tests are not sensitive enough to detect CWD prions in body fluids or in animals with subclinical infection (18). This is important when testing animals in very early stages of infection with low concentrations of CWD prions. Despite advancements in technology and creation of other prion assays, most of the unknowns surrounding CWD and other prion diseases remain because of testing limitations.

Although ELISA and IHC are not validated food safety tests and therefore cannot guarantee that an animal is completely free of CWD prions, they are the best available options to reduce human exposure. Current data on prion concentrations in different cervid body tissues suggest that an ELISA and/or IHC result of “not detected” from the tested central nervous system or lymphoid tissue provides reassurance that CWD prions are likely not present in skeletal muscle (19–21). Continuing to use ELISA and IHC is essential until improved, validated assays are available.

Diagnosing human prion diseases is also a major challenge. Substantial variation in early clinical presentation can complicate the accurate diagnosis of prion diseases. Other diagnostic obstacles include the wide range of disease duration and lack of physician familiarity with the condition (22). Antemortem tests can help guide disease diagnosis, including cerebrospinal fluid tests (e.g., real-time quaking-induced conversion [RT-QuIC], tau, and 14-3-3), brain magnetic resonance imaging, and electroencephalography. Suspected cases can be confirmed via postmortem tests, including neuropathologic examination, Western blotting, and IHC. Confirming human prion disease cases through autopsy is critical as CWD exposure grows. Continued use of autopsy will clarify the risk of CWD transmission and could identify novel prion diseases that may have occurred via secondary transmission through other animals. Additionally, examination of lymphoreticular tissues in individuals exposed to CWD prions could highlight any potential risk to the blood supply, as seen with vCJD. Sustaining a robust human prion disease surveillance system at the state level is critical.

## CWD: OPERATING IN THE UNKNOWN

Risk-based public health policies and practices are essential as CWD exposure grows. Numerous challenges, however, hinder progress. Multiple studies have attempted to assess the integrity of the CWD species barrier between cervids and humans. *In vitro* species barrier studies expose human PrP^C^ to CWD prions outside a living organism and measure whether or not the human PrP^C^ are converted. While most studies found CWD prions capable of converting human PrP^C^, the efficiency of conversion was low or relied on protein modifications (23).

Several studies using nonhuman primates have provided conflicting results. Squirrel monkeys appear to be highly susceptible to CWD infection following either oral or intracerebral exposure (24, 25). Cynomolgus macaques have also been used as an animal model because of their genetic similarity to humans. Before 2017, evidence of prion infection among cynomolgus macaques exposed orally or intracerebrally to CWD prions had not been documented (25–27). However, recent research involving four cynomolgus macaques supports the potential for infection (28, 29). Two monkeys showing signs of prion infection had a CWD-contaminated steel wire implanted in their brain, while the other two orally consumed skeletal muscle tissue, approximately equivalent to a human eating one 7-ounce steak per month, from asymptomatic, CWD-positive deer. These latter possible infections are most concerning, as that route of exposure is the most realistic for humans. Although the study is ongoing and has not been subjected to peer review, results reported to date cause concern over robustness of the CWD species barrier.

Major limitations in both *in vitro* and *in vivo* studies make the assessment of risk to human health challenging. *In vitro* studies can provide timely insight into the species barrier, but they cannot represent the complexities that exist within a living organism. Additionally, variations in methodology complicate comparisons of results (23). *In vivo* studies of prion diseases are expensive and lengthy owing to the ecology of prion diseases and their prolonged incubation. While *in vivo* studies can address factors such as the influence of exposure routes, prion interactions taking place in animals might not equate to those in humans. This difference is important in prion studies, where one polymorphic variation in the host PRNP gene can play a role in susceptibility to infection (30). While *in vitro* and *in vivo* species barrier studies are important, they have not provided definitive answers regarding whether CWD will infect humans or other animals, such as bovines.

Available data suggest that the risk of CWD transmission to humans is low but not zero (7). However, evidence also suggests the species barrier is not static. Factors such as different CWD strains emerging, cervid species, and polymorphic variations can play key roles in interspecies transmission (31).

Recent animal studies show that CWD prions can adapt following serial passage, resulting in new strains. Prion strains possess slight differences in their conformation and transmission properties, and different prion strains can be identified through both their biological and biochemical features in the host. For example, various strains appear to have noticeably different incubation periods or cause unique clinical symptoms (32, 33). Evidence also suggests that emerging CWD strains could have broader host ranges and higher zoonotic potential (34, 35).

Although researchers have believed that CWD originated in the American West, scientists now speculate that the disease also is emerging sporadically across the globe via multiple strains. Recent cases among cervids in Norway and Sweden and experimental evidence demonstrating that the cervid PrP^C^ may be more prone to misfolding into new prion strains support this theory (29, 36–38). Spontaneous conversion of cervid PrP^C^ to CWD prions suggests that continued evolution of CWD prions may increase its zoonotic potential, but further research is needed to confirm this. Regardless, the CWD species barrier between cervids and humans may not be fixed, and the risk for interspecies transmission may increase as CWD continues to spread and adapt.

*In vitro* and *in vivo* experiments designed to assess strain adaptation and species barriers have concentrated on prions found in the central nervous system, typically the brain. Using tissue from the central nervous system is biologically plausible because the overwhelming majority of prion infectivity can be found there. However, prions can be found in many diverse extraneural tissues, including muscle, blood, and lymphoid organs (39–42). Experiments determining whether these tissues harbor or replicate prions distinct from neural prions had not been investigated until Beringue and Laude found greater permissiveness through prion transmission barriers of extraneural prions than neural prions (43). These data suggest that prions found in peripheral tissues may hold more zoonotic potential than prions found in neural tissue.

A lack of assurance of an absolute species barrier necessitates a preventive public health approach. Since 1997, the World Health Organization has recommended that agents of any prion disease should not enter the human food chain (11). Likewise, the U.S. Centers for Disease Control and Prevention, Health Canada, and numerous state health and wildlife agencies recommend that people should not consume the meat of a CWD-positive animal (11). Despite these recommendations, decisions about handling CWD-positive animals remain with the hunters. Some choose to eat the meat regardless. For example, the Wisconsin Department of Health Services maintains a list of individuals who have consumed CWD-positive animals, with nearly 1,000 people agreeing to participate in the state’s long-term disease surveillance program (44).

## A CALL TO ACTION

Despite the best efforts of wildlife agencies and other organizations to combat CWD, a unified approach has not been developed. A comprehensive strategy, with national leadership and support, is needed to address this important public health risk. The following immediate steps need to be taken to mitigate the unnecessary risk of human exposure and advance our understanding of transmission risk: (i) investing additional resources in CWD research, (ii) enacting and enforcing mandatory CWD testing of dead or harvested cervids in all areas of endemicity, (iii) improving management practices to prevent CWD transmission among cervids, and (iv) heightened surveillance of human prion diseases to determine if CWD is transmissible to humans.

### Investing in CWD research.

Investment in CWD research, which includes support for disease surveillance and management, is an essential first step for controlling CWD. CWD was declared a national emergency in 2001, and Congress began appropriating federal funds to support CWD research, surveillance, and management at that time (17). However, a major decrease in federal funding occurred after 2011, and states largely have been responsible for funding CWD efforts since that time. Federal support for cervid health dropped from $14.3 million in 2011 to $1.9 million in 2012 (45). Annual federal funding has remained modest since that time, making it difficult for state wildlife, animal health, and public health agencies to appropriately address this problem. By restoring adequate federal support, these agencies can conduct critical animal and human disease surveillance, enhance hunter education, and better develop and conduct comprehensive CWD management plans.

Improved diagnostic testing is a key research need. While use of existing tools is important, development of a validated, rapid, and reliable test for CWD that can be conducted in the field could have significant public health impact. Current testing methods are labor-intensive, and results can be delayed, increasing the risk of exposure to hunters. Additionally, infected animals could enter meat-processing plants before determination of their disease status, introducing the potential for cross-contamination. Increased CWD research investment will also help answer unresolved questions regarding disease pathways and mechanisms, ultimately providing needed detail on the risk of transmission between cervids and the potential threat to humans. Finally, research is needed on development of effective vaccines or treatments to protect cervids and potentially decrease CWD spread.

### Enhancing mandatory CWD cervid testing.

Policies and strategies for CWD testing are decided by state agencies. CWD testing is voluntary for hunters in some states. Numerous other states have established mandatory testing policies, but not across all geographic areas with documented CWD in cervids. Inadequate testing policies can result in CWD surveillance gaps. For example, Wisconsin’s Department of Natural Resources (DNR) has established surveillance areas around detections of CWD among wild cervid populations and within captive cervid facilities (46). However, testing remains voluntary in these areas, with the Wisconsin DNR relying on hunters to submit samples. Despite the Wisconsin DNR offering CWD testing to hunters in surveillance areas at no cost, only 5% of Wisconsin’s 336,464 deer harvested in 2018 were tested (47, 48). Furthermore, only 4,925 of the 23,441 deer harvested in four Wisconsin counties (Dane, Iowa, Richland, and Sauk) where CWD is most established were tested in 2018, with 894 (18%) testing positive. More than 18,500 deer harvested in these four Wisconsin counties were not tested for CWD, suggesting that the meat from at least 3,000 CWD-positive animals was consumed, given the previously determined prevalence. Until gaps in CWD surveillance are addressed, the extent of CWD in cervid populations will remain unknown and people will unknowingly consume prion-infected meat.

Mandatory testing of killed or dead cervids in all areas of endemicity and establishing associated monitoring systems that can be shared across states would provide more accurate data for wildlife managers and public policy makers. Improved surveillance would also foster more efficient responses and help prevent disease spread among cervids. Additionally, implementing mandatory testing in areas of endemicity results in hunters knowing if consumption of their hunted cervid will expose them to CWD prions. Targeted and professionally developed education strategies can aim to reduce the consumption of CWD-positive animals, making mandatory testing in areas of endemicity an important first step in preventing human exposure to CWD prions. However, there remains a critical need for further interventions, since some individuals continue to knowingly consume meat from cervids that are CWD positive.

### Improving cervid management.

The Association of Fish and Wildlife Agencies (AFWA) has published the best CWD management practices based on current science (16). The practices AFWA addresses, including the movement of live cervids, carcass disposal, and feeding/baiting, need to be implemented by states as soon as possible, backed by sufficient resources. Science-based and enforced regulation of captive cervid facilities is essential to prevent transmission of CWD within such herds and subsequently reduce risk of transmission to wildlife populations. Without comprehensive, effective changes in wildlife management and the captive cervid industry, the nearly $40 billion annual contribution of wild cervid hunting in the United States is under threat. Although proposed policies may be a burden for some hunters and cervid farmers, following best management practices will likely improve the health of wild and farm-raised cervids.

## CONCLUSION

Available data indicate that the incidence of CWD in cervids is increasing and that the potential exists for transmission to humans and subsequent human disease. Given the long incubation period of prion-associated conditions, improving public health measures now to prevent human exposure to CWD prions and to further understand the potential risk to humans may reduce the likelihood of a BSE-like event in the years to come.
